# Interaction of APOE e4 and poor glycemic control predicts white matter hyperintensity growth from 73 to 76

**DOI:** 10.1016/j.neurobiolaging.2017.02.014

**Published:** 2017-06

**Authors:** Simon R. Cox, Stuart J. Ritchie, David Alexander Dickie, Alison Pattie, Natalie A. Royle, Janie Corley, Benjamin S. Aribisala, Sarah E. Harris, Maria Valdés Hernández, Alan J. Gow, Susana Muñoz Maniega, John M. Starr, Mark E. Bastin, Joanna M. Wardlaw, Ian J. Deary

**Affiliations:** a Centre for Cognitive Ageing and Cognitive Epidemiology, University of Edinburgh, Edinburgh, UK; b Department of Psychology, University of Edinburgh, Edinburgh, UK; c Scottish Imaging Network, a Platform for Scientific Excellence (SINAPSE) Collaboration, Edinburgh, UK; d Brain Research Imaging Centre, Neuroimaging Sciences, Centre for Clinical Brain Sciences, University of Edinburgh, Edinburgh, UK; e Department of Computer Science, Lagos State University, Lagos, Nigeria; f Centre for Genomic and Experimental Medicine, MRC Institute of Genetics & Molecular Medicine, University of Edinburgh, Edinburgh, UK; g Department of Psychology, School of Life Sciences, Heriot-Watt University, Edinburgh, UK; h Alzheimer Scotland Dementia Research Centre, University of Edinburgh, UK

**Keywords:** White matter, Aging, Brain MRI, APOE, Vascular risk, Longitudinal

## Abstract

We examined whether apolipoprotein E (*APOE*) status interacts with vascular risk factors (VRFs) to predict the progression of white matter hyperintensities (WMHs) on brain MRI scans over a specific period of life in older age when the risk of dementia increases. At age 73 years, baseline VRFs were assessed via self-reported history of diabetes, hypertension, smoking, and hypercholesterolemia, and via objective measures of blood HbA1c, body mass index, diastolic and systolic blood pressure, and blood high-density lipoprotein to total cholesterol (HDL) ratio. *APOE* e4 allele was coded as either present or absent. WMH progression was measured on MRI over 3 years in 434 older adults, in a same-year-of-birth cohort. *APOE* e4 carriers with either a self-reported diagnosis of diabetes (*β* = 0.160, *p* = 0.002) or higher glycated hemoglobin levels (*β* = 0.114, *p* = 0.014) exhibited greater WMH progression, and the former survived correction for multiple testing. All other *APOE*-VRF interactions were nonsignificant (*β*_interaction_ < 0.056, *p* > 0.228). The results suggest that carrying the *APOE* “risk” e4 allele increases the risk of greater age-related WMH progression over the early part of the eighth decade of life, when combined with poorer glycemic control. The interaction effect was robust to co-occurring VRFs, suggesting a possible target for mitigating brain and cognitive aging at this age.

## 1 Introduction

Brain white matter hyperintensities (WMHs) are a prevalent MRI feature of healthy and pathologic states in older age and are associated with important life outcomes (Debette and Markus, 2010, Kloppenborg et al., 2014, Lee et al., 2016, Longstreth et al., 1996) and vascular risk factors (VRFs) such as hypertension, diabetes, smoking, obesity, and hypercholesterolemia (de Leeuw et al., 2004, Dufouil et al., 2001, Prins and Scheltens, 2015, Wang et al., 2015, Wardlaw et al., 2014). Compared with cross-sectional WMH volumetric measures, WMH progression shows a stronger relation with important age-related functional changes (Prins and Scheltens, 2015, van Dijk et al., 2008), and it is possible that different VRFs are relevant at different ages (Zhang et al., 2015). Hence, longitudinal studies with a narrow age range are essential to identify the most pertinent VRFs at specific periods in life.

Alongside evidence of VRF-WMH associations, WMH heritability is estimated at 55%–80% (Atwood et al., 2004, Carmelli et al., 1998, Turner et al., 2004). Variation in the apolipoprotein E (*APOE*) gene (which delivers essential lipids to neurons and is linked to accelerated neurodegeneration Bu, 2009, Mahley and Rall, 2000), is a plausible candidate to explain some of this heritability. Possession of the e4 “risk” allele is associated with more WMHs and poorer white matter microstructure in older adults (Brickman et al., 2014, Heise et al., 2011) and greater cognitive decline (Bangen et al., 2013, Schiepers et al., 2012, Wisdom et al., 2011). However, *APOE* was not associated with cross-sectional WMH load in 2 large studies (Fornage et al., 2011, Paternoster et al., 2009). Discrepancies between heritability estimates of complex disease and the risk explained by the common genetic variants might be due to interactions with VRFs (Manolio et al., 2009). Prior examinations of *APOE*-VRF interactions involved cross-sectional white matter measures across participants of a broad age range, yielding inconsistent results (de Leeuw et al., 2004, Foley et al., 2014, Wang et al., 2015).

The current study is the first, to our knowledge, to test the interactions between *APOE* e4 status and VRFs on longitudinal WMH progression.

## 2 Materials and methods

### 2.1 Participants

Members of the Lothian Birth Cohort 1936 were initially 1091 older adults, most of whom took part in the Scottish Mental Survey 1947 when aged 11 years and were living in the Edinburgh and Lothian areas of Scotland at the start of Wave 1 of testing (at ∼70 years of age in 2004–2007; Deary et al., 2007, Deary et al., 2012). All were White Caucasian and reported no diagnosis of dementia at baseline. 488 participants attended brain MRI scans at both Waves 2 and 3 (mean ages 72.65 and 76.36 years). Of these, 434 provided blood for genotyping at Wave 1 and VRF data at Wave 2. The Multi-Centre Research Ethics Committee for Scotland (MREC/01/0/56), the Scotland A Research Ethics Committee (07/MRE00/58) and the Lothian Research Ethics Committee (LREC/2003/2/29) approved use of the human subjects in this study; all participants provided written informed consent and these have been kept on file.

### 2.2 APOE status

Genotyping on the 2 polymorphic sites (rs7412 and rs429358) that account for the e2, e3, and e4 alleles (Wenham et al., 1991) was performed on genomic DNA, isolated from whole blood, using TaqMan technology by the Wellcome Trust Clinical Research Facility Genetics Core, Western General Hospital, Edinburgh. An exact test (Web Reference 1) confirmed that the APOE genotypes were in Hardy-Weinberg equilibrium (*p* = 0.657).

### 2.3 Vascular risk factors

The World Heart Federation,, UK National Health Service,, Framingham Heart Study,, and the British Heart Foundation (Web References 2-5) overlap in their identification of diabetes, hypertension, smoking, high body mass index (BMI), and hypercholesterolemia as important VRFs and are pertinent to WMH burden in older age (de Leeuw et al., 2004, Dufouil et al., 2001, Prins and Scheltens, 2015, Wang et al., 2015, Wardlaw et al., 2014). VRFs were assessed during a cognitive and physical testing appointment at age 73 years. During a medical interview, diagnosis of diabetes, hypertension, smoking (current, ex, or never), and hypercholesterolemia were reported. Objective VRFs were BMI (weight kg/height m^2^), pulse pressure (difference between average systolic and diastolic blood pressure, taken over 6 consecutive measurements, 3 sitting and 3 standing, from an Omron 705IT monitor), blood glycated hemoglobin (HbA1c using a Menarini HA8160 analyser), and the ratio of high-density lipoprotein to total cholesterol (HDL ratio; Milián et al., 2009).

### 2.4 MRI acquisition and processing

Whole brain MRI was performed on each participant at ages 73 and 76 years using the same scanning protocol in the same scanner (GE Signa Horizon 1.5 T HDx; Milwaukee, WI, USA). T1-, T2-, T2*-, and FLAIR-weighted sequences were coregistered at a resolution of 1 × 1 × 2 mm. A semi-automated multispectral fusion method (Valdés Hernández et al., 2010) combined these sequences to measure the intracranial and WMH volumes. WMHs were explicitly defined as punctate, focal, or diffuse lesions in all subcortical regions (Wardlaw et al., 2013). All segmented images were visually examined for accuracy on anonymized scans to correct errors and ensure that infarcts (including lacunar infarcts, n = 3 at baseline in this sample) and enlarged perivascular spaces were excluded from WMH masks. Full details are available in an open-access protocol (Wardlaw et al., 2011).

### 2.5 Statistical analysis

WMH volumes at age 73 and 76 years were expressed as a proportion of intracranial and corrected for age in days on the date of image acquisition. Residuals of the regression between log WMH volume at Waves 2 (IV) and 3 (DV) were derived to index WMH volume change (ΔWMH) over a mean of 3.71 (SD = 0.27) years. Using the “sem” function in the “lavaan” package (v.0.5–22) in R v3.2.2, we used ΔWMH as the dependent variable in models with VRFs and *APOE* status (e4 allele present or absent) as predictors, alongside an interaction term (APOE e4 status × VRF), for each VRF. We used full information maximum likelihood estimation to reduce bias due to missingness, under the assumption of “missing at random” (Rubin, 1976), including the following “auxiliary” variables (previously used to model dropout in this cohort; Ritchie et al., 2016): age 11 IQ, years of education, father's and own social class, Scottish Index of Multiple Deprivation at recruitment, forced expiratory volume over 1 second, 6-meter walk, hand grip strength (best of 6; 3 with each hand), a binary variable indicating a self-reported diagnosis of dementia or MMSE score <24 at any wave of testing (n = 22). In instances where interaction terms were significant, coincident VRFs were inserted as covariates. Standardized *β*s, corrected for false discovery rate (FDR) are reported throughout. Variance inflation factors (VIFs) among VRFs was ascertained using “vif” in the “usdm” package for R. In a supplementary, a posteriori analysis, we further explored interactions between *APOE* status and both HbA1c and diabetes. We split *APOE* e4 and e2/3 groups by diabetes diagnosis or high/low HbA1c level (median split at 5.59 DCCT), visualized, and tested (*t*-tests) group differences in WMH progression.

## 3 Results

**Fig. 1:**
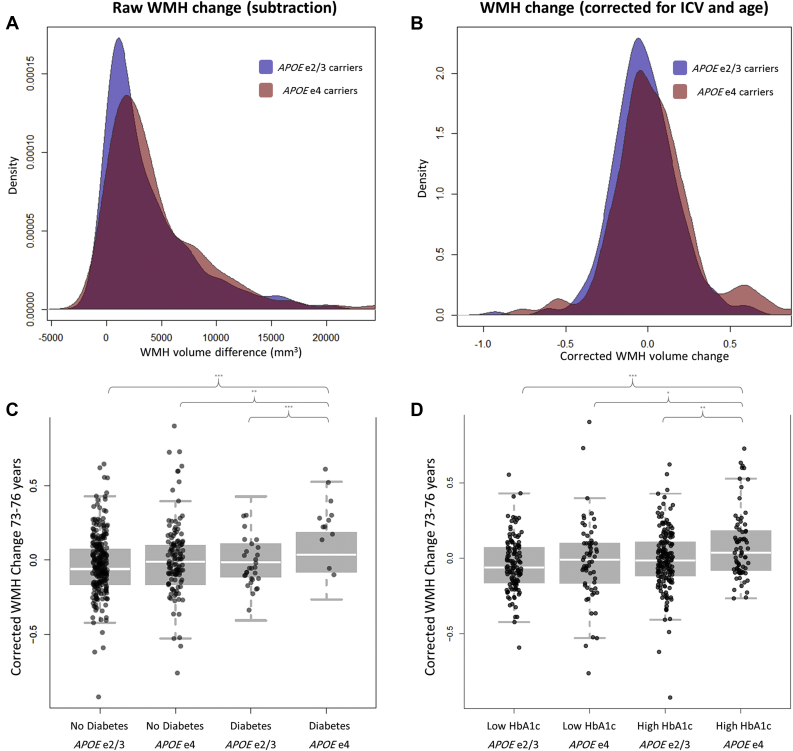
Three-year white matter hyperintensity progression and group differences by APOE status and either diabetes or HbA1c. Kernel density plots of white matter hyperintensity progression split by *APOE* status are shown for (A) raw uncorrected change in mm^3^, calculated by subtraction and (B) WMH change calculated as the residuals of the regression between age 73 and 76 volumes, which were each log transformed and corrected for ICV and age at scan. A score of zero therefore denotes average WMH change in this and subsequent panels. The group differences in corrected WMH change split by APOE status and (C) self-reported diabetes diagnosis and (D) HbA1c level are shown (lower panels). Groups created from the whole sample based on a median split of HbA1c (Low range 4.4–5.59 DCCT, High range 5.60–8.9 DCCT), and *APOE* e4 status (carriers vs. noncarriers). Brackets indicate significant group differences at ^∗^*p* < 0.05, ^∗∗^*p* < 0.01, ^∗∗∗^*p* < 0.001. Bold horizontal lines represent group means and vertical lines denote standard error.

Participant characteristics are shown in Table S1, and density plots of the raw and corrected measures of WMH change are shown in Fig. 1. There was a significant increase in corrected WMH volume in under 4 years (*t* (865.34) = 3.685, *p* < 0.001) in the total sample. Associations among the VRFs were modest (Table S2) and showed low variance inflation factor (all <1.87). Exploratory factor analysis provided no basis to extract 1 (*χ*^2^ = 164.8, *p* = 8.85 × 10^−25^; 18.4% of the variance) or 2 (*χ*^2^ = 59.96, *p* = 5.33 × 10^−8^; 27.9% of the variance) factors of general vascular risk. A total of 136 participants were *APOE* e4 carriers, who were not significantly different to non-e4 carriers in terms of age, cross-sectional WMH volume at either wave, male:female ratio, MMSE score at Wave 2, or VRF status. *APOE* e4 carriers did, however, show a significantly lower MMSE score at Wave 3 (*p* = 0.020, Table S1).

Effects of VRFs on WMH volume change—without reference to *APOE—*have previously been reported in this sample (Dickie et al., 2016). The main effects are therefore reported here for illustrative purposes, but the focus of the current analyses is specifically to examine VRF-*APOE* interactions on WMH progression. Main effects of *APOE* and VRFs on WMH volume change, and their interaction, are reported in Table 1. *APOE* e4 carriers and those with a lower HDL ratio showed nominally greater WMH progression than non-e4 carriers (*β* = 0.118, *p* = 0.014 and *β* = −0.101, *p* = 0.034, respectively), but these did not survive FDR correction.

Participants with either a self-reported diagnosis of diabetes (*β* = 0.160, *p* = 0.002) or higher HbA1c (*β* = 0.114, *p* = 0.014) showed greater WMH progression, but only for *APOE* e4 carriers. Only the interaction between *APOE* e4 and diabetes survived FDR correction. Correcting these relationships for other, co-occuring VRFs (BMI, hypertension, and hypercholesterolemia based on Table S2), did not substantially alter the interaction effects for diabetes (*β* = 0.167, *p* < 0.001) and HbA1c (*β* = 0.118, *p* = 0.008). Further group-wise analysis showed that participants with diabetes, or high HbA1c, who carried the *APOE* e4 allele exhibited significantly greater change in WMH volume progression over 3.7 years than all other groups (all *t*-values >2.34; Fig. 1).

## 4 Discussion

The present study tested whether *APOE* status interacted with important VRFs in contributing to WMH volume change from age 73–76 years in a large group of community-dwelling older adults with a narrow age range. Our novel results suggest that WMH growth was greater in those with diabetes and a higher HbA1c, but only in those who possessed an *APOE* e4 allele, though only the former interaction survived FDR correction.

The finding that *APOE* e4 is associated with WMH progression extends previous cross-sectional associations between *APOE* status and WMH volume in older age (Brickman et al., 2014, de Leeuw et al., 2004, Wang et al., 2015). Rather than being an index of the degree to which *APOE* status relates to accumulated WMH burden up to a specific (single) point of assessment, the current data suggest that carrying the e4 allele continues to influence the degree of WMH progression at this specific age. In isolation, diabetes was not associated with WMH progression (consistent with previous studies; Prins and Scheltens, 2015), but, importantly, we found that those with a current diagnosis of diabetes showed significantly greater WMH progression, but only if they carried the *APOE* e4 allele. The effect size was similar for an objective measure of glycemic control, but this finding did not survive FDR correction. These findings are consistent with prior associations between diabetes risk–including glycemic control in “pre-diabetes”—and APOE allele status for cognitive decline and dementia (Dore et al., 2009, Irie et al., 2008, Peila et al., 2002, Roriz-Filho et al., 2009). Taken together, this evidence provides support for the hypothesis that, as with other complex diseases, genetic interactions are important for understanding relationships between VRFs and WMH progression in older age, and identifying potential targets for amelioration of brain aging and dementia.

The duration, level, and success of medical intervention for VRF-control in the current sample (such as statins for hypercholesterolemia, antihypertensive, or antidiabetic medication) are unknown, and may affect these findings. We also relied on self-report for information on a variety of clinical diagnoses. Nevertheless, the use of blood biomarkers such as HDL ratio and HbA1c allow objective measures, independent of self-report for some VRFs. More importantly, although the current total sample is relatively large, the proportion of individuals with both diabetes and the e4 allele was small, thus the specific diabetes-*APOE* interaction effect should be tested independently. However, the presence of the same interaction (with similar magnitude) with HbA1c might ostensibly corroborate the biological plausibility of this result. Having only 2 waves of data makes our results more susceptible to artifacts such as regression to the mean (see Nesselroade et al., 1980) and other sources of bias such as measurement error. Our use of full information maximum likelihood in this relatively large sample can only guard against these possibilities to some degree. Finally, some sample characteristics limit the confidence with which these findings can be generalized to groups of different ages, ethnicities, and pathologic states. Nevertheless, these sample characteristics can also be viewed as study strengths, as they attenuate important potential confounds of age, ethnicity, and the effect of infarcts on WMH measurement, offering valuable insights into specific age and interactions that might influence the progression of structural brain damage.

The reduction of average blood glucose levels is identified here as a potentially meaningful predictor of WMH progression in the mid-1970s among *APOE* e4 carriers. This work requires replication in other larger prospective imaging studies, and also at different ages to examine whether, and which, interactions contribute to life-course WMH volume changes. Furthermore, *APOE* is only one of the many potential genetic contributors to WMH burden in older age. Its consistent association with dementia and cognitive decline make it an obvious candidate for interaction studies, but WMH risk is likely to be polygenic (Lopez et al., 2015), and future studies should examine other genetic-VRF interactions for predicting WMH volume and progression.

## Disclosure statement

The authors declare no potential conflicts of interest.

## Figures and Tables

**Table 1:** Main effects and interaction effects of APOE status in regressions between vascular risk factor (VRF) and white matter hyperintensity change

Predictors of ΔWMH	[VRF]		[VRF] × *APOE*	
	*β*	*p*	*β*	*p*
APOE	0.114	0.015		
HbA1c	0.105	0.034	0.114	0.014
Diabetes	0.089	0.091	0.160	0.002
Body mass index	−0.017	0.744	−0.015	0.757
Pulse pressure	−0.067	0.149	−0.020	0.679
Hypertension	−0.037	0.429	0.004	0.938
Smoking	0.043	0.403	−0.024	0.633
HDL ratio	−0.108	0.025	0.028	0.590
Hypercholesterolemia	0.002	0.974	−0.056	0.228

Standardized betas reported.

Key: Diabetes, self-reported diagnosis of diabetes; HbA1c, glycated hemoglobin; HDL ratio, ratio of high-density lipoprotein to total cholesterol; Smoking, never–ex-smoker–current; ΔWMH, white matter hyperintensity volume change.

## References

[bib1] Atwood L.D., Wold P.A., Heard-Costa N.L., Massaro J.M., Beiser A., D'Agostino R.B., DeCarli C. (2004). Genetic variation in white matter hyperintensity volume in the Framingham study. Stroke.

[bib2] Bangen K.J., Beiser A., Delano-Wood L., Nation D.A., Lamar M., Libon D.J., Bondi M.W., Seshadri S., Wolf P.A., Au R. (2013). APOE genotype modifies the relationship between midlife vascular risk factors and later cognitive decline. J. Stroke Cerebrovasc. Dis..

[bib3] Brickman A.M., Schupf N., Manly J.J., Stern Y., Luchsinger J.A., Provenzano F.A., Narkhede A., Razlighi Q., Collins-Praino L., Artero S., Akbaraly T.N., Ritchie K., Mayeux R., Portet F. (2014). APOE e4 and risk for Alzheimer's disease: do regionally distributed white matter hyperintensities play a role?. Alzheimers Dement.

[bib4] British Heart Foundation Heart health – Risk Factors. https://www.bhf.org.uk/heart-health/risk-factors.

[bib5] Bu G. (2009). Apolipoprotein E and its receptors in Alzheimer's disease: pathways, pathogenesis and therapy. Nat. Rev. Neurosci..

[bib6] Carmelli D., DeCarli C., Swan G.E., Jack L.M., Reed T., Wolf P.A., Miller B.L. (1998). Evidence for genetic variance in white matter hyperintensity volume in normal elderly male twins. Stroke.

[bib7] de Leeuw F.-E., Richard F., de Groot J.C., van Duijn C.M., Hofman A., Van Gijn J., Breteler M.M. (2004). Interaction between hypertension, apoE, and cerebral white matter lesions. Stroke.

[bib8] Deary I.J., Gow A.J., Pattie A., Starr J.M. (2012). Cohort profile: the Lothian birth cohorts of 1921 and 1936. Int. J. Epidemiol..

[bib9] Deary I.J., Gow A.J., Taylor M.D., Corley J., Brett C., Wilson V., Campbell H., Whalley L.J., Visscher P.M., Porteous D.J., Starr J.M. (2007). The Lothian Birth Cohort 1936: a study to examine influences on cognitive ageing from age 11 to age 70 and beyond. BMC Geriatr..

[bib10] Debette S., Markus H.S. (2010). The clinical importance of white matter hyperintensities on brain magnetic resonance imaging: systematic review and meta-analysis. BMJ.

[bib11] Dickie D.A., Ritchie S.J., Cox S.R., Sakka E., Royle N.A., Aribisala B.S., Valdés Hernández M.C., Muñoz Maniega S., Pattie A., Corley J., Starr J.M., Bastin M.E., Deary I.J., Wardlaw J.M. (2016). Vascular risk factors and progression of white matter hyperintensities in the Lothian Birth Cohort 1936. Neurobiol. Aging.

[bib12] Dore G.A., Elias M.F., Robbins M.A., Elias P.K., Nagy Z. (2009). Presence of the APOE e4 allele modifies the relationship between type 2 diabetes and cognitive performance: the Maine-Syracuse Study. Diabetalogica.

[bib13] Dufouil C., de Kersaint-Gilly A., Basnçon V., Levy C., Auffray E., Brunnereau L., Alpérovitch A., Tzourio C. (2001). Longitudinal study of blood pressure and white matter hyperintensities: the EVA MRI cohort. Neurology.

[bib14] Foley J.M., Salat D.H., Stricker N.H., Zink T.A., Grande L.J., McGlinchey R.E., Milberg W.P., Leritz E.C. (2014). Interactive effects of apolipoprotein E4 and diabetes risk on later myelinating white matter regions in neurologically healthy older aged adults. Am. J. Alzheimers Dis. Other Demen..

[bib15] Fornage M., Debette S., Bis J.C., Schmidt H., Ikram M.A., Dfouil C., Sigurdsson S., Lumley T., DeStafano A.L., Fazekas F., Vrooman H.A., Shibata D.K., Maillard P., Zijdenbos A., Smith A.V., Gudnason H., de Boer R., Cushman M., Mazoyer B., Heiss G., Vernooij M.W., Enzinger C., Glazer N.L., Beiser A., Knopman D.S., Cavalieri M., Neissen W.J., Harris T.B., Petrovic K., Lopez O.L., Au R., Lambert J.C., Hofman A., Gottesman R.F., Garcia M., Heckbert S.R., Atwood L.D., Catellier D.J., Uitterlinden A.G., Yang Q., Smith N.L., Aspelund T., Romero J.R., Rice K., Taylor K.D., Nalls M.A., Rotter J.I., Sharrett R., van Duijn C.M., Amouyel P., Wolf P.A., Gudnason V., van der Lugt A., Boerwinkle E., Psaty B.M., Seshardi S., Tzourio C., Breteler M.M., Mosley T.H., Schmidt R., Longstreth W.T., DeCarli C., Launer L.J. (2011). Genome-wide association studies of cerebral white matter lesion burden: the CHARGE consortium. Ann. Neurol..

[bib16] Framingham Heart Study Cardiovascular Disease. https://www.framinghamheartstudy.org/risk-functions/cardiovascular-disease/10-year-risk.php.

[bib17] Hardy-weinberg calculator website (2012). http://www.had2know.com/academics/hardy-weinberg-equilibrium-calculator-3-alleles.html.

[bib18] Heise V., Filippini N., Ebmeier K.P., Mackay C.E. (2011). The APOE e4 allele modulates brain white matter integrity in healthy adults. Mol. Psychiatry.

[bib19] Irie F., Fitzpatrick A.L., Lopez O.L., Kuller L.H., Peila R., Newman A.B., Launer L.J. (2008). Enhanced risk for Alzhiemer disease in persons with type 2 diabetes and APOE e4. Arch. Neurol..

[bib20] Kloppenborg R.P., Nederkoorn P.J., Geerlings M.I., van den Berg E. (2014). Presence and progression of white matter hyperintensities and cognition: a meta-analysis. Neurology.

[bib21] Lee S., Viquar F., Zimmerman M.E., Narkhede A., Tosto G., Benzinger T.L.S., Marcus D.S., Fagan A.M., Goate A., Fox N.C., Cairns N.J., Holtzman D.M., Buckles V., Ghetti B., McDade E., Martins R.N., Saykin A.J., Masters C.L., Ringman J.M., Ryan N.S., Forster S., Laske C., Schofield P.R., Sperling R.A., Salloway S., Correia S., Jack C., Weiner M., Bateman R.J., Morris J.C., Mayeux R., Brickman A.M. (2016). White matter hyperintensities are a core feature of Alzheimer's disease: Evidence from the Dominantly Inherited Alzheimer Network. Ann. Neurol..

[bib22] Longstreth W.T., Manolio T.A., Arnold A., Burke G.L., Bryan N., Jungries C.A., Enright P.L., O'Leary D., Fried L. (1996). Clinical correlates of white matter findings on cranial magnetic resonance imaging of 3301 elderly people: the Cardiovascular Health Study. Stroke.

[bib23] Lopez L.M., Hill W.D., Harris S.E., Valdés Hernández M., Muñoz Maniega S., Bastin M.E., Bailey E., Smith C., McBride M., McClure J., Graham D., Dominiczak A., Yang Q., Fornage M., Ikram M.A., Debette S., Launer L., Bis J.C., Schmidt R., Seshadri S., Porteous D.J., Starr J.M., Deary I.J., Wardlaw J.M. (2015). Genes from a translational analysis support a multifactorial nature of white matter hyperintensities. Stroke.

[bib24] Mahley R.W., Rall S.C. (2000). Apolipoprotein E: far more than a lipid transport protein. Ann. Rev. Genomics Hum. Genet..

[bib25] Manolio T.A., Collins F.S., Cox N.J., Goldstein D.B., Hindorff L.A., Hunter D.J., McCarth M.I., Ramos E.M., Cardon L.R., Chakravarti A., Cho J.H., Guttmacher A.E., Kong A., Kruglyak L., Mardis E., Rotimi C.N., Slatkin M., Valle D., Whittemore A.S., Boehnke M., Clark A.G., Eichler E.E., Gibson G., Haines J.L., Mackay T.F., McCarroll S.A., Visscher P.M. (2009). Finding the missing heritability of complex diseases. Nature.

[bib26] Milián J., Pintó X., Muñoz A., Zuñiga M., Rubriés-Prat J., Pallardo L.F., Masana L., Mangas A., Hernández-Mijares A., González-Santos P., Ascaso J.F., Pedro-Botet J. (2009). Lipoprotein ratios: physiological significance and clinical usefulness in cardiovascular prevention. Vasc. Health Risk Manag..

[bib27] Nesselroade J.R., Stigler S.M., Baltes P.B. (1980). Regression toward the mean and the study of change. Psychol. Bull.

[bib28] Paternoster L., Chen W., Sudlow C.L.M. (2009). Genetic determinants of white matter hyperintensities on brain scans: a systematic assessment of 19 candidate gene polymorphisms in 46 studies in 19000 subjects. Stroke.

[bib29] Peila R., Rodriguez B.L., Launer L.J., Honolulu-Asia Aging Study (2002). Type 2 diabetes, APOE gene, and the risk for dementia and related pathologies: the Honolulu-Asia Aging Study. Diabetes.

[bib30] Prins N.D., Scheltens P. (2015). White matter hyperintensities, cognitive impairments and dementia: an update. Nat. Rev. Neurol..

[bib31] Ritchie S.J., Tucker-Drob ETm Cox S.R., Corley J., Dykiert D., Redmond P., Pattie A., Taylor A.M., Sibbet R., Starr J.M., Deary I.J. (2016). Predictors of ageing-related decline across multiple cognitive functions. Intelligence.

[bib32] Roriz-Filho J.S., Sá-Roriz T.M., Rosset I., Camozzato A.L., Santos A.C., Chaves M.L., Moriguti J.C., Roriz-Cruz M. (2009). (Pre)diabetes, brain aging, and cognition. Biochim. Biophys. Acta.

[bib46] Rubin D.B. (1976). Inference and missing data. Biometrika.

[bib33] Schiepers O.J., Harris S.E., Gow A.J., Pattie A., Brett C.E., Starr J.M., Deary I.J. (2012). APOE e4 predicts age-related cognitive decline in the ninth decade: a longitudinal follow-up of the Lothian Birth Cohort 1921. Mol. Psychiatry.

[bib34] Turner S.T., Jack C.R., Fornage M., Mosley T.H., Kardia S.L., Boerwinkle E., de Andrade M. (2004). Heritability of leukoariosis in hypertensive sibships. Hypertension.

[bib35] UK National Health Service Cardiovascular disease – Risk factors. http://www.nhs.uk/Conditions/cardiovascular-disease/Pages/risk-factors.aspx.

[bib36] Valdés Hernández M.C., Ferguson K.J., Chappell F.M., Wardlaw J.M. (2010). New multispectral MRI data fusion technique for white matter lesion segmentation: method and comparison with thresholding in FLAIR images. Eur. Radiol..

[bib37] van Dijk E.J., Prins N.D., Vrooman H.A., Hofman A., Koudstaal P.J., Breteler M.M. (2008). Progression of cerebral small vessel disease in relation to risk factors and cognitive consequences: Rotterdam Scan study. Stroke.

[bib38] Wang R., Fratiglioni L., Laukka E.J., Lövdén M., Kalpouzos G., Keller L., Graff C., Salami A., Bäckman L., Qui C. (2015). Effects of vascular risk factors and APOE e4 on white matter integrity and cognitive decline. Neurology.

[bib39] Wardlaw J.M., Allerhand M., Doubal F.N., Valdés Hernández M.C., Morris Z., Gow A.J., Bastin M.E., Starr J.M., Dennis M.S., Deary I.J. (2014). Vascular risk factors, large-artery atheroma, and brain white matter hyperintensities. Neurology.

[bib40] Wardlaw J.M., Bastin M.E., Valdés Hernández M.C., Muñoz Maniega S., Royle N.A., Morris Z., Clayden J.D., Sandeman E.M., Eadie E., Murray C., Starr J.M., Deary I.J. (2011). Brain aging, cognition in youth and old age and vascular disease in the Lothian Birth Cohort 1936: rationale, design and methodology of the imaging protocol. Int. J. Stroke.

[bib41] Wardlaw J.M., Smith E.E., Biessels G.J., Cordonnier C., Fezekas F., Frayne R., Lindley R.I., O'Brien J.T., Barkhof F., Benavente O.R., Black S.E., Brayne C., Breteler M., Chabriat H., DeCarli C., de Leeuw F.E., Doubal F., Deuring M., Fox N.C., Greenberg S., Hacinski V., Kilimann I., Mok V., Oostenbrugge R.V., Pantoni L., Speck O., Stephan B.C., Teipel S., Viswanathan A., Werring D., Chen C., Smith C., van Buchem M., Norrving B., Gorelick P.B., Dichgans M., Standards for Reporting Vascular change on nEuroimaging (STRIVE v1) (2013). Neuroimaging standards for research into small vessel disease and its contribution to ageing and neurodegeneration. Lancet Neurol..

[bib42] Wenham P.R., Price W.M., Blandell G. (1991). Apolipoprotein E genotyping by one-stage PCR. Lancet.

[bib43] Wisdom N.M., Callahan J.L., Hawkins K.A. (2011). The effects of apolipoprotein E on non-impaired cognitive functioning: a meta-analysis. Neurobiol. Aging.

[bib44] World Heart Federation Cardiovascular Disease Risk Factors. http://www.world-heart-federation.org/press/fact-sheets/cardiovascular-disease-risk-factors/.

[bib45] Zhang C.R., Cloonan L., Fitzpatrick K.M., Kanakis A.S., Ayres A.M., Furie K.L., Rosand J., Rost N.S. (2015). Determinants of white matter hyperintensity burden differ at the extremes of ages of ischemic stroke onset. J. Stroke Cerebrovasc. Dis..

