# Supplementary material for: Interaction of APOE e4 and poor glycemic control predicts white matter hyperintensity growth from 73 to 76
Source: Neurobiol Aging. 2017 Jun;54:54–8. doi: 10.1016/j.neurobiolaging.2017.02.014 (PMC5407886; doi:10.1016/j.neurobiolaging.2017.02.014)
Supplement: Tables S1 and S2 [file mmc1.docx]

**Supplementary Material**

*Table S1.* Participant characteristics

|  | **Full Sample** | ***APOE* e4** | |  |
| --- | --- | --- | --- | --- |
|  |  | **No** | **Yes** | ***p*** |
| N | 434 | 298 | 136 |  |
| Sex (M:F) | 238:196 | 164:134 | 74:62 | 0.986 |
| Wave 2 Age (yrs) | 72.65 (0.70) | 72.65 (0.67) | 72.66 (0.77) | 0.880 |
| Wave 3 Age (yrs) | 76.36 (0.64) | 76.35 (0.61) | 76.39 (0.69) | 0.504 |
| Wave 2 MMSE (/30) | 28.88 (1.32) | 28.91 (1.27) | 28.83(1.44) | 0.603 |
| Wave 3 MMSE (/30) | 28.54 (1.83) | 28.69 (1.64) | 28.21 (2.17) | 0.020 |
| WMH 73 (cm^3^) | 11.79 (11.30) | 11.83 (11.88) | 11.69 (9.93) | 0.901 |
| WMH 76 (cm^3^) | 15.77 (14.45) | 15.57 (15.08) | 16.21 (13.00) | 0.653 |
| HbA1c (DCCT) 73 | 5.74 (0.62) | 5.75 (0.61) | 5.73 (0.62) | 0.727 |
| Diabetes (Y:N) | 41:393 | 29:269 | 12:124 | 0.902 |
| Body Mass Index | 27.78 (4.21) | 27.74 (4.06) | 27.84 (4.55) | 0.830 |
| Systolic BP | 146.51 (18.31) | 146.51 (18.41) | 146.52 (18.13) | 0.994 |
| Diastolic BP | 79.75 (9.20) | 79.97 (9.30) | 79.27 (8.99) | 0.457 |
| Pulse Pressure | 66.76 (15.12) | 66.54 (14.90) | 67.25 (15.64) | 0.654 |
| Hypertension (Y:N) | 215:219 | 152:146 | 63:73 | 0.423 |
| Smoking 73 | 218:188:28 | 143:132:23 | 75:56:5 | 0.171 |
| HDL ratio | 3.77 (1.1) | 3.74 (1.1) | 3.83 (1.1) | 0.415 |
| High Cholesterol (Y:N) | 182:252 | 124:174 | 58:78 | 0.922 |

*Note.* Measures given are Mean (SD) for scalar data, or as ratios for ordinal data. WMH = white matter hyperintensity volume (uncorrected), Hb1Ac = glycated haemoglobin, DCCT = Diabetes Control and Complications Trial units, Diabetes (Y = self-reported diagnosis), Smoking (never : ex-smoker : current), HDL ratio = ratio of high density lipoprotein to total cholesterol, MMSE = Mini-Mental State Examination. *p*-values are outcomes of Welch’s *t*-test between *APOE* e4 carriers and non-carriers are reported (except Chi Squared for ordinal and binary data). Of those with a self-reported diagnosis, the following numbers reported taking medication for diabetes (n = 27), hypertension (n = 201) and high cholesterol (n = 130).

*Table S2.* Correlations among vascular risk factors.

|  | **HbA1c** | **Diabetes** | **BMI** | **PP** | **HighBP** | **Smoking** | **HDL** |
| --- | --- | --- | --- | --- | --- | --- | --- |
| **HbA1c** | - |  |  |  |  |  |  |
| **Diabetes** | 0.636^c^ | - |  |  |  |  |  |
| **BMI** | 0.222^c^ | 0.160^b^ | - |  |  |  |  |
| **PP** | -0.078^a^ | -0.055 | -0.060 | - |  |  |  |
| **HighBP** | 0.155^b^ | 0.137^b^ | 0.208^c^ | -0.107^b^ | - |  |  |
| **Smoking** | 0.056 | 0.051 | 0.008 | -0.025 | -0.007 | - |  |
| **HDL** | 0.030 | -0.083 | 0.163^b^ | -0.034 | -0.060 | 0.046 | - |
| **HiCHOL** | 0.155^b^ | 0.188^c^ | 0.124^a^ | -0.025 | 0.344^c^ | 0.013 | -0.179^c^ |

*Note.* Pearson’s *r* reported, except Spearman’s *rho* for ordinal and point-biserial *r*_pb_ for binary data. HbA1c = glycated haemoglobin (3log transformed), Diabetes = self-reported diagnosis of diabetes, BMI =body mass index, PP = pulse pressure, HighBP = hypertension, Smoking (never : ex-smoker : current), HDL = ratio of high density lipoprotein to total cholesterol, HiCHOL = hypercholesterolemia. ^a^ *p* < 0.05, ^b^ *p* < 0.01, ^c^ *p* < 0.001.
